# **A comprehensive tool in recycling plant-waste of** *Gossypium barbadense* L **agricultural and industrial waste extracts containing gossy**pin** and gossypol: hepatoprotective, anti-inflammatory and antioxidant effects**

**DOI:** 10.1186/s13007-024-01181-8

**Published:** 2024-04-17

**Authors:** Mona A. Mohammed, Nagat M. Amer, Heba M. I. Abdallah, Mai S. Saleh

**Affiliations:** 1 Medicinal and Aromatic Plants Research Department, Pharmaceutical and Drug Industries Research Institute, National Research Centre, Dokki, Giza, Egypt; 2 Environmental and Occupational Medicine Department, Environment and Climate Change Research Institute, National Research Centre, Dokki, Giza, Egypt; 3 Pharmacology Department, Medical Research and Clinical Studies Institute, National Research Centre, Dokki, Giza, Egypt

**Keywords:** Solid waste treatment, Gossypol, Gossypin, Anti-inflammatory, Hepatoprotective, Antioxidant, Bioactive components

## Abstract

Improper management of agricultural and industrial cotton wastes causes environmental pollution and worsens the climate change challenge. Green recycling of cotton could contribute to a circular economy. One of the economic values of cotton wastes lies in their bioactive components. Two types of cotton wastes—agricultural and industrial—of the species *Gossypium barbadense* L. Giza 95 were targeted in the current study, aiming to maximize their medicinal value and investigate the anti-inflammatory, hepatoprotective, and antioxidant activities of their phytochemical extracts. Phytochemical extraction was performed using different solvents extraction. An anti-inflammatory effect was tested in carrageenan-induced acute edema in a rat paw model. A carbon tetrachloride chronic model of liver injury was used for the assessment of hepatoprotective potential. Liver enzymes (AST and ALT), oxidative stress markers (MDA and GSH), inflammatory biomarkers (C-reactive protein), and histopathological features were investigated. As a result, ethyl acetate proved to be the solvent of best choice to extract the gossypin polyphenolics, where the extracted amount reached 14,826.2 µg/g, followed by butanol (8751.4 µg/g extract). The chloroform (CHCL_3_) fraction showed the highest amounts of gossypol (190.7 µg/g extract), followed by petroleum ether. Cotton waste’s composition analysis showed a wide range of components, including 33 metabolites such as gossypetin, polyphenolics, and other metabolites that possess therapeutic effects. Both chloroform extract and industrial waste extracts showed superior anti-inflammatory and hepatoprotective effects in comparison to other extracts. All tested extracts (ethyl acetate, chloroform, and industrial waste) showed proper antioxidant activities.

**Graphical Abstract:**

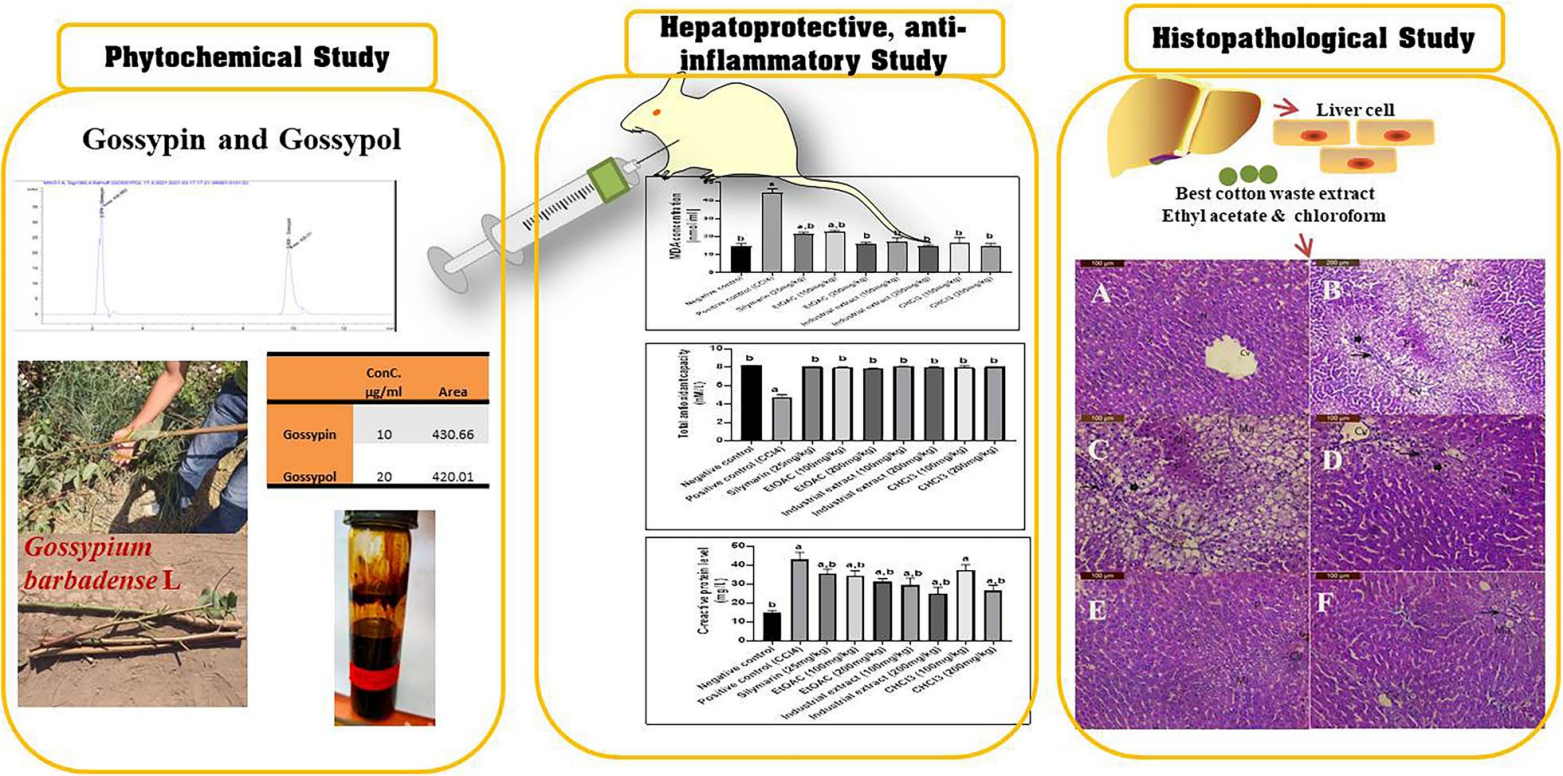

**Supplementary Information:**

The online version contains supplementary material available at 10.1186/s13007-024-01181-8.

## Background

The management process of solid waste is rendered a major public health issue that invites a big deal of environmental concern especially in urban areas of developing countries. Unfortunately, the improper solid waste management may cause a substantial negative environmental impact that includes health and safety problems and prevalence of diseases that are normally associated with environmental pollution [1].

Solid waste management is a major challenge for many countries, for example the public sector in Egypt is in short of providing the adequate services for solid waste treatment due to the limited existing regulations and the inappropriate local taxation system [2]. As a consequence the illegal disposal of domestic and industrial waste is still the common practice [3–5].

Cotton fiber is considered to be one of the most important natural textile fiber worldwide. It is ranked the third among the total textile market [6] and in Egypt, the Egyptian cotton (*Gossypium barbadense* L.) is considered as an economically important cash crop. The agricultural waste in Egypt is unfortunately misused and is subjected to either burning or improper disposing. This manipulations usually results in the over consumption of potential valuable resources in addition to a non-desirable increase in the Green House Gases emission. Such a situation is inviting the search for new and innovative solutions that allow an eco-friendly and sustainable waste disposal to cotton textile solid wastes [7].

Cotton textile waste comes in two varieties: pre-consumer and post-consumer, through mechanical and chemical methods. The quality requirements of recycling and reusing cotton in clothing and fabric applications have been the subject of numerous studies. More work is needed to meet the quality requirements for additional applications [6]. The present research work is concerned with the pre-consumer cotton waste. Normally, after cultivation of cotton, the waste is left in the field where it decomposes. Afterwards, the ginning process takes place where cotton fiber is separated from the lint. The lint is used in the spinning process and the non-lint fraction, is separated into about 85% as cotton seeds used to obtain oil or stock feed, and a remaining 15% that is classified as a waste product. In most cases, this waste product is used to produce fertilizers, oil spill clean-up, or in ethanol manufacturing. While the cotton fiber is prepared for spinning by the industrial manufacturer with 4.7% separated as waste.

The appropriate utilization of agro-industrial matrices necessitates the generation or extraction of bioactive compounds employing environmentally friendly methods rather than traditional processes, followed by the optimization of process conditions. In this particular context, biological processes emerge as noteworthy, as they possess the ability to augment the production, extraction, and application of constituents from agro-industrial matrices in a more appealing manner. Owing to their selectivity, biological strategies offer certain benefits, such as the generation of extracts characterized by superior quality and bioactivity, as well as low toxicity. The present study furnishes an outline of the primary bioactive constituents discovered in agro-industrial by-products by using solvent extracts rather than biological methods. Additionally, we provide information to enhance the utilization of these bioactive compounds in the extracts in the realms of pharmaceutical industries [8, 9].

The cotton extract possess a lot of valuable bioactive components. Such bioactive components have been reported to show various activities that include analgesic, cardioprotective effect [10], stopping bleeding [11], and curing chronic bronchitis. Potent biologically active components in cotton like the flavonoids gossypetin and gossypin, the sesquiterpene aldehyde gossypol, and the trisaccharide gossypose are responsible for these biological and therapeutic effects. For example, gossypol proved inhibitory effect on different cancer diseases, gossypose showed immunomodulatory effects, while gossypin became known for its bactericidal activities. However, less attention is paid to the medicinal value of cotton metabolites with a great desire for more profound and comprehensive investigation of the economic potential for bioactive components that could be extracted from cotton textile solid waste [8, 9].

In such context and for maximizing the economic benefit of benefiting from both agricultural and industrial cotton waste, the current study aims to investigate the anti-inflammatory, hepatoprotective, and antioxidant effects of different cotton waste solvent extracts. Phytochemical characterization of extracts that shows biological active moieties with quantitative estimation of gossypin and gossypol is another crucial objective to draw more comprehensive conclusions.

## Materials and methods

### Chemicals and reagents

Carrageenan, indomethacin, Carbon tetrachloride (CCl4) (purity ≥ 99.0%), and formaldhyde were obtained from Sigma-Aldrich (St. Louis, MO, USA). Indomethacin was used as a standard anti-inflammatory and analgesic drug.

### Phytochemical study

#### Plant materials Egyptian cotton wastes

The present study was carried out on two pre-consumer cotton textile solid wastes; the agricultural waste (non-lint fraction after removing the seeds), and the industrial waste produced after spinning of cotton fibers. The agricultural waste of the Egyptian cotton; *Gossypium barbadense* L. Giza 95 was obtained from a farm at Beni-suef governorate belonging to the Cotton Research Institute, Cairo, Egypt, and was identified in their laboratories by Dr. Abdel Naser, Deputy Director. Whereas, the industrial waste was obtained from a private factory for the weaving and spinning of cotton in Giza, Egypt.

#### Plant extraction

Extraction is the first step to separate the desired natural products from the raw materials. successive extraction methods include different solvent extraction using petroleum ether, chloroform, ethylacetate, butanol, and water where pressing and sublimation are experienced according to Hamed et al.,[12].

### Pytochemical investigation

#### Phytochemical screening

The following screening procedures have been applied;

1. *Detection of volatile substances* according to Guenther [13]. Ten grams of the samples were subjected to water distillation (500 mL water) using Clevenger's apparatus. The distillation was continued for 3 h after boiling.
2. *Detection of carbohydrates and glycosides*, 0.5 g of each extract sample was diluted in 5 mL of 50% ethanol. The presence of carbohydrates and glycosides in the methanolic extract were detected by using drops from α- naphthol sulphuric acid reagent as reported by Lewis and Smith [14]. Formation of a red or dull violet color at the interphase of the two layers was a positive test.
3. *Detection of tannins*, The method of Shellard [15] has been used for the detection of tannins in extracts samples by the addition of few drops of 10% ferric chloride in ethanol reagent. The change of color to bluish black or precipitate, indicates the presence of tannins.
4. *Detection of alkaloid***,** 1 mL of the alcoholic extract filtrate was mixed with 2 mL of Dragendoff's reagent; a turbid orange color indicates the presence of alkaloids. The confirmation test was done using Mayer's reagent; hence appearance of yellow precipitate indicates the presence of the alkaloids [16].
5. *Detection of flavonoids*, 1 mL of the alcoholic extracts with 1% HCl acid overnight and filtered. Then, the filtrate were rendered alkaline with NaOH (1 M) and the formation of yellow color indicates the probable presence of flavonoids according to Trease and Evans, [17].
6. *Detection of the presence of flavanones and flavonol*, magnesium/HCl were added to methanolic extract, a red color formation indicates the presence of flavanones and flavonol according to Shinoda [18].
7. *Detection of saponins*, about 0.5 g of the dry extracts was macerated in 20 mL distilled water and the filtrate was shaken vigorously. A persisting froth for about 30 min. indicates the possible presence of saponins according to Shellard, [15].
8. *Detection of sterols and triterpenes*, the presence of unsaturated sterols and triterpenes was detected using acetic acid anhydride (0.3 mL)–sulphuric acid (0.5 mL) reagent and observed for the formation of a brown ring at the junction of two layers. Green coloration of the upper layer and the formation of deep red color in the lower layer would indicate a positive test for steroids and triterpenoids respectively, as reported by Hanson [19].

### Quantitative and qualitative estimation of gossypin and gossypol in cotton wastes by HPLC and LC–ESI–MS/MS technique

The HPLC system in use was an HP 1100 chromatograph (Agilent Technologies, Palo Alto, CA, USA) equipped with an auto-sampler (G1329B), quaternary pump, and a diode array detector [20]. The measurements were integrated by Chemstation chromatographic software interfaced to a personal computer. The analytical column was ZORBAX Eclipse XDB C-18 column (15 cm × 4.6 mm I.D., 5 µm, USA) according to Cai et al. [21].

LC–ESI–MS/MS analysis was performed using an Exion LCAC system for separation and SCIEX Triple Quad 5500 + MS/MS system equipped with an ESI for detection. The separation was performed with an Ascentis^®^ C18 Column (4.6 × 150 mm, 3 µm). The mobile phases consisted of A: 0.1% formic acid; B: acetonitrile (LC grade) [22]. The mobile phase gradient was programmed as follows: 10% B at 0–1 min, 10–90% B from 1 to 33 min, 90% B from 33 to 37 min, 10% at 37.1, 10% from 37.1 to 40 min. The flow rate was 0.7 ml/min and the injection volume was 10 µl. For MS/MS analysis, negative ionization mode was applied with a scan (EMS-IDA-EPI) from 100 to 1000 Da for MS1 with the following parameters: curtain gas: 25 psi; IonSpray voltage: − 4500; source temperature: 500 °C; ion source gas 1 and 2 were 45 psi and from 50 to 800 Da for MS2 with a declustering potential: − 80; collision energy: − 35; collision energy spread: 15. Compounds’ identification was performed using MS-DIAL software version 4.70 [23, 24].

### Antioxidants activity (DPPH^•−^ & ABTS^•+^ method)

DPPH^**•−**^ and ABTS^**•+**^ free radical-scavenging activity method was adopted to measure the in vitro antioxidant activity of two main extracts of the total industrial waste and total agricultural waste and it’s their six fractions: petroleum ether, chloroform, ethylacetate, butanolic, water and precipitate water; at different concentrations (30, 20, 10, 5, 2.5, 1.5, 1 and 0.5 μg/mL) of the eight extracts. Ascorbic acid, trolox, Gossypin and Gossypol were used as standards for reference data. All determinations were carried out in triplicates are illustrated in Fig. (4).The radical scavenging model for antioxidant activity, using 1,1-diphenyl-2-picrylhydrazyl (DPPH, 250 mM), was performed according to Mohammed et al. [25].

ABTS was dissolved in water to a 7 mM concentration. ABTS radical cation (ABTS  +) was produced by reacting ABTS stock solution with 2.45 mM potassium persulfate. Appropriate solvent blanks were run in each assay according to Dinkova-Kostova et al. [26]. The percentage inhibition of the DPPH and ABTS radicals was calculated using the following formula:

$$\% {\text{Inhibittion}}\,{ = }\,\left[ {{{\left( {{\text{A}}\,\,\,{\text{control - a}}\,\,\,{\text{sample}}} \right)} \mathord{\left/ {\vphantom {{\left( {{\text{A}}\,\,\,{\text{control - a}}\,\,\,{\text{sample}}} \right)} {{\text{A}}\,\,\,\,{\text{control}}}}} \right. \kern-0pt} {{\text{A}}\,\,\,\,{\text{control}}}}} \right]\, \times 100$$

where A is the absorbance at 517 nm in DPPH and 734 nm in ABTS.

### Pharmacological study

#### Animals

Male Wistar rats weighing between 180 and 200 g were utilized for the experiment. They were obtained from the National Research Center's animal house colony and kept in a standard environment with 12 h light/dark cycles, a standard pellet diet, and relative humidity of 55 to 10%. The present study complies with local and national guidelines, as it was done in accordance with the guide for care and use of laboratory animals and obtained ethics committee approval certificate from National Research Centre ethics committee numbered [Ethical approval no: 2317/1022021]. Experiments were performed according to the National Regulations of Animal Welfare and the Institutional Animal Ethical Committee (IAEC) and are reported in accordance with Animal Research: Reporting of In Vivo Experiments (ARRIVE) guidelines.

The rats were put to sleep with ketamine (50 mg/kg) for animal anaesthesia during blood sampling. Formaldehyde was used for fixation of postmortem tissues dissected for histopathologic examination.

#### Acute toxicity study

Total, CHCL_3_, EtOAc, and industrial extracts were given orally to male albino mice of both sexes in graduated doses up to 4 g/kg. The equal amount of distilled water was given to the control group. After 24 h, the animals were checked for mortality. Additionally, mice’s skin, hair, respiratory, circulatory, and behavioral patterns were examined for any changes. This was performed by dissolving the extracts in physiological saline.

#### Investigation of anti-inflammatory activity of cotton waste extracts in rats

The carrageenan-induced rat hind paw edema test was used to assess the extract's acute anti-inflammatory activity [27]. Sixty-six adult male Wistar rats were divided into 11 groups, with six rats in each group. First group was given normal saline solution (10 mL/kg, p.o.) and represents the control group. Indomethacin was provided to the second group. (10 mg/kg). The third group received gossypin (10 mg/kg) [28]. Groups 4–11 received the different cotton extracts; ethyl acetate, total extract, industrial sample, and choloform extracts; respectively. Each extract was given at two dose levels, 100 and 200 mg/kg; respectively (according to acute toxicity study). All of the animals received an 0.1 mL injection of 1% (v/v) carrageenan solution in saline at the subplanter region of the right hind paw one hour after receiving the extracts orally. Before administering carrageenan, the volume of each rat's paws was measured using a digital caliber, and then it was checked hourly for the next four hours. The following formula was used to determine each group's percentage of edema:

$${\text{Edema}}\,\,{\text{(E)}}\,\,{\text{\% }}\,{ = }\left( {{\text{V}}_{{\text{t}}} - {\text{V}}_{{0}} } \right) \times {100}$$

where; Vo is the volume (in milliliters) prior to the carrageenan injection, and Vt is the volume at time t following the injection (mL).

### Investigation of hepatoprotective and antioxidant effects of cotton extracts against CCl4-induced chronic hepatotoxicity in rats

Nine groups of 54 male Wistar rats (six rats per each group) were used in the current study. The first group was considered a control negative and was given olive oil (1 mL/kg, p.o.) as a single loading dose. After 1 week, rats were administered olive oil (0.5 mL/kg, p.o.) twice/week and saline (0.5 mL/150 g, p.o.) every day for 4 weeks. The second group (control positive) was given CCl_4_ to induce chronic hepatotoxicity. At the beginning, a single loading dose of CCl_4_ (1 mL/kg, p.o.) was administered to rats. Then, after one week, CCl_4_ (0.5 mL/kg, p.o.) was then administered for four weeks [29, 30]. Groups 3–8 were injected with CCl_4_, given the same dose as the second group, and treated with cotton extracts; ethyl acetate, industrial, and CHCl_3_ extracts. Silymarin (25 mg/kg), the standard hepatoprotective drug, was given to the 9th group. Each extract was administered orally at two doses of 100 and 200 mg/kg, respectively (according to acute toxicity study). All treatments were administered daily for 4 weeks. Twenty-four hours after last drug administration, animals were anaesthetized, blood samples from the retro-orbital venous plexus were taken, and blood was allowed to clot [31].

Serum was separated using a cooling centrifuge by centrifugation at 3000 rpm for 20 min at 4 °C (Sigma Laborzentrifugen GmbH, Germany). The liver function was evaluated through measuring serum aspartate transaminase (AST), alanine transaminase (ALT), and oxidative stress indicators (malonedialdehyde (MDA) and total antioxidants (TAC)). C-reactive protein was measured for anti-inflammatory activity using commercially available kits. Then, rats were sacrificed, and livers were removed out and submerged in ice-cold saline solution. For histological analysis, samples from each liver major lobe of 3 animals were fixed in 8% saline-buffered formalin.

### Biochemical analysis

#### Hepatotoxicity indices

Serum AST and ALT were determined using available kits (Biodiagnostic, Egypt) according to the method of Reitman and Frankel [32]. Principally, the transfer of the amino group from glutamic acid to oxalacetic acid and pyruvic acid is catalyzed by the glutamic transaminase enzymes serum glutamic pyruvic acid (GPT) and serum glutamic oxalacetic acid (GOT) in reversible processes. The transaminase activity is determined by reaction with 2.4 dinitrophenylhydrazine (DNPH) in an alkaline solution. This reaction is proportional to the quantity of oxaloacetate pyruvate generated during a specific period of time.

#### Oxidative stress markers

Commercially available kits (Biodiagnostic, Giza, Egypt) were used to measure lipid peroxides (as "MDA") and total antioxidant capacity spectrophotometrically by following manufacturer's instructions. Ohkawa et al. [33] method was used to quantify lipid peroxides in liver homogenates, and Koracevic et al. [34] method to measure Total antioxidant capacity (TAC).

Lipid peroxidation was calculated as the MDA content, which when combined with thiobarbituric acid (TBA) in an acidic media yields a pink TBA-reactive product that can be detected by spectrophotometry at 534 nm. On the other hand, the sample's antioxidants were used to measure the total antioxidative capacity (TCA) by removing a certain quantity of additional hydrogen peroxide. An enzymatic process that yielded a colorful product is used to quantify the colorimetric residual hydrogen peroxide at 505 nm.

#### Determination of c-reactive protein

C-reactive protein was assessed by enzyme-linked immunoassay (ELISA) technique according to manufacturer's instructions ((MyBioSource, Inc., San Diego, USA). Sandwich enzyme immunoassay is the test principle used. Standards or samples were added with a target-specific biotin-conjugated antibody to the corresponding microtiter plate wells which has been pre-coated with a target-specific antibody. Each microplate well was then filled with horseradish peroxidase (HRP)-conjugated avidin, and the mixture were incubated. Only the wells containing the target, biotin-conjugated antibody, and enzyme-conjugated avidin would change color when the TMB substrate solution was introduced. A sulphuric acid solution was added to stop the enzyme–substrate reaction, and the color change was detected spectrophotometrically at 450 nm. Next, the O.D. of the samples and the target concentration in the samples were compared.

#### Histopathological examinations

Liver samples, were fixed in formalin, treated with a standard alcohol and xylol blend, embedded in paraffin, and sectioned into 5 mm thick sections. Haematoxylin and eosin (H and E) staining were applied to the sections in order to examine the histopathological alteration. Images were taken at the National Research Center’s pathology lab using an Olympus CX41 light microscope and a SC100 digital camera connected to a computer system.

### Statistical analysis

Statistical analysis was done using SPSS version 22. The degree of variability in results was expressed as the mean ± standard error of the mean (SEM). Data were calculated by one-way analysis of variance (ANOVA) followed by Tukey multiple comparisons. The significance level was set at P < 0.05.

## Results and discussions

### Phytochemical Study

#### Solvent extraction of cotton waste

**Fig. 1:**
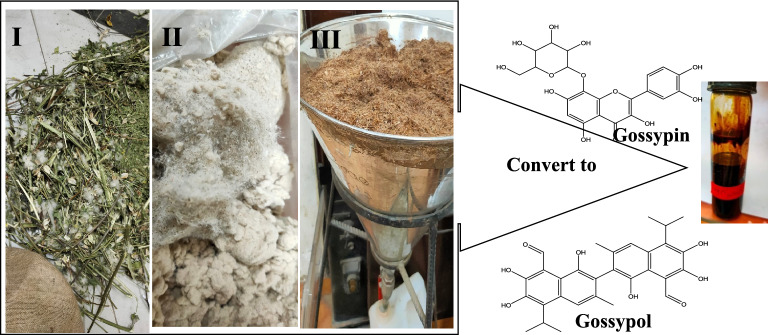
I Agriculture waste, **II** Industrial waste, **III** percolator of agriculture waste extraction

A total of 2537 g of powder from the agricultural waste, and 250 g of powder from the industrial waste were separately extracted by methanol (80%), and soaked at room temperature to obtain the total extract for both wastes. Both alcoholic extracts were concentrated under reduced pressure at 45 °C using rotary evaporator, and a yield of 240.811 g of residue was obtained from agricultural waste and 12.22 g was obtained from industrial waste [35]. The crude residue from agricultural waste was suspended in 500 ml water, left for the overnight, and successively partitioned (V/V) with 1L pet. ether (40–60%) was gave (40.066 g), 1 L methylene chloride (8.5544 g), 1 L ethyl acetate (2.2945 g) and 1 L n-butanol (19.1818 g). The water residue and precipitate extract were then obtained by weight as 150.459 g, and 20.256 g, respectively (Fig. 1).

#### Phytochemical screening

The results of the preliminary phytochemical screening of cotton waste extracts are shown in Table (1). The results show the presence of flavonoids, carbohydrates, tannins, triterpenoids and/or steroids, alkaloids, and saponin. Volatile oil was not detected in all of the eight samples. These results agree with the results obtained by Kumar et al. (2013) [36] who stated the presence of flavonoids, carbohydrates, saponins, phenols, coumarins, and Cardiac glycosides. However alkaloid, triterpenes, and tannins were detected in our samples.

**Table 1:** Phytochemical screening of agriculture and industrial wastes

Groups	Agricultural waste							Industerial waste
	Total Agri_waste	Pet. ether	CHCL_3_	EtOAc	Butanol	H2O	Pre_H_2_O	
Volatile Oils	**–**	**–**	**–**	**–**	**–**	**–**	**–**	**–**
Carbohydrate	** + + + **	**–**	**–**	** + **	** + + + **	** + + + **	** + **	** + + + **
Tannins	** + + + **	**–**	**–**	**–**	** + + + **	** + + + **	** + **	** + + **
Flavonoids, NaOH	** + + + **	**–**	** + **	** + + **	** + + + **	** + + **	** + **	** + + + **
Flavonoids (Shinoda test)	** + + + **	**-**	** + **	** + + **	** + + + **	** + + **	** + + **	** + + + **
Saponin	** + + + **	** + + + **	**-**	** + + **	** + + + **	** + + + **	**–**	** + + **
Sterol and / or triterpenes	** + + **	** + + + **	** + + + **	**–**	**–**	**–**	**–**	** + **
Coumarins	** + + + **	** + + + **	** + + **	** + + **	** + + **	** + **	**–**	** + + **
Alkaloids	** + + **	** + **	** + + **	** + + **	** + **	**–**	**–**	**–**

(+ +), ( +), and (−) refer to high, low, and absente amounts, respectively

#### Quantitative estimation of gossypin and gossypol in cotton waste extracts as determined by HPLC technique

Determination of gossypol (polyphenolic aldehyde) and gossypin (a glycosyloxyflavone) was done using single different binary gradients for the analysis. Gossypol and gossypin in the seven different agricultural extracts, and the industrial extract followed the following HPLC binary gradient program that was found to be optimum: 0–10 min isocratic with 80:20 aqueous: acetonitrile 0.1% content. The flow rate was 1 mL min and total run time 15 min. The retention time for gossypol was found to be 9.808 min and gossypin 2.374 min. (Additional file 1: Fig. S1). This method has proven to be highly reproducible. The maximum detection of both; gossypin and gossypol was detected in chloroform extract as 190.7 ug, and 3539.3 µg, respectively. This method in use is a simple, fast, and cost-effective method for quantification of gossypin and gossypol.

Ethyl acetate showed to be the solvent of best choice to extract the gossypin polyphenolics where the amount obtained reached 14,826.2 µg/g which is higher than that extracted by butanol (8751.4 µg/g) as shown in (Table 2). Chloroform fraction contains the highest amounts of gossypol (190 µg/g extract), followed by petroleum ether that goes in agreement with Clark [37].

**Table 2:** Quantitative of gossypin and gossypol in agriculture and industrial Cotton waste by HPLC technique

Extracts (conc. µg/g)	Gossypin	Gossypol
1. Total Agricultural waste	5837.4	0.0
2. Petroleum. Ether	1107.1	105.9
3. Chloroform	3539.3	190.7
4. Ethylacetate	14,826.2	0.0
5. Butanol	8751.4	0.0
6. Water	811.3	0.0
7. Precipitate _H_2_O	264.5	0.0
8. Industrial waste	998.4	0.0

The possible causes of the various solvents’ differing extraction efficiency; the solubility revealed that gossypol was present in chloroform in an aldehyde-aldehyde state in just 5 days. After that, for 45 days, the aldehyde–aldehyde and lactol–lactol tautomeric forms coexisted and kept the solution stable. Gossypol mostly existed in aldehyde–aldehyde form when it was dissolved in methanol. The freshly made methanol solution included very little lactol-lactol. It was discovered that gossypol only existed in the lactol-lactol combination for 45 days. Gossypol was present in dimethyl sulfoxide in the aldehyde–aldehyde, lactol–lactol, and ketol–ketol forms. Throughout the 45 day period, there was a competitive connection between the aldehyde–aldehyde and lactol–lactol forms. Going over all the conditions and solvents examined, it was discovered that gossypol was extremely stable in chloroform. These findings are in agreement with Wang et al. [38].

Only flavonoids that contain chiral carbon atoms in their molecular structure exhibit optical activity. Flavonoids that are not bound to other compounds are typically soluble in various organic solvents such as methanol, ethanol, ethyl acetate, chloroform, and ether. However, gossypin, a specific flavonoid, has a higher solubility in water and a lower solubility in anhydrous organic solvents. This unique property poses challenges in obtaining gossypin that is completely free from mineral matter, thereby hindering the acquisition of accurate analytical data. Despite this limitation, satisfactory results were obtained from the analysis of gossypin's carbon and hydrogen composition, as well as the estimation of the products derived from acid hydrolysis, including glucose and gossypetin. These findings support the proposition that gossypin can be represented by a monoglucoside formula [39] Additional file 2: Figure S2.

The choice of the proper solvents; CHCL_3_ for gossypol, and EtOAc for gossypin may depend on the solubility of the phenolic nature of these components [37]. These findings are in agreement with Meyer et al. [40] who indicated that the methanol extract is the best solvent to be used for the quantitative HPLC of these phenolics. The industrial waste showed to contain only gossypin and free from gossypol because this waste contain some seed as appear in (Fig. 1c) that findings are agreements with European Food Safety Authority [41]

#### LC–ESI–MS/MS technique of cotton waste extracts

Cotton waste extracts contain a wide range of 33 primary metabolites that function mostly as essential factors in cell biosynthetic pathways. The primary metabolites were identified based on comparison of mass spectra to the MS-Dial library. In this study, we successfully identified by LC–MS a list of various secondary metabolites in two cotton waste extracts which are: polyphenolic acids (Gossypetin, Laricitrin, Ellagic acid, Quinic acid, Isorhamnetin, Vergosin, Deoxyhemigossypol, Kaempferol, and Astragalin), some plant hormones (Dihydrozeatin, and Estrone), vitamins (Threonic acid, and Pantothenic acid), amino acid and other compounds (Table 3). Secondary metabolites are providing protective mechanisms with an expected hepatoprotective effect. The chemical profiles share compositional similarity with Park et al. [42].

**Table 3:** LC–MS metabolites of agriculture waste and industrial waste

No	RT (min)	Metabolite name	m/z [M-H]^−^	Mass fragments	Molecular formula	Agricuture waste	Industrial waste	Gaussian	S/N	Refs.
1	1.50	2-Hydroxyisobutyric acid	103.10	76	C_4_H_8_O_3_		*	0.868897	6.012494	MS-dial
2	1.63	N-Methyllysine	159.25	130/111	C_7_H_16_N_2_O_2_	*	*	0.656398	6.071359	MS-dial
3	1.92	Gluconic acid	195.20	–	C_6_H_12_O_7_		*	0.961515	65.01656	[42]
4	2.10	Cysteic acid	167.90	152	C_3_H_7_NO_5_S		*	0.719606	26.75691	[43]
5	2.41	4-Isopropylbenzoic acid	162.95	–	C_10_H_12_O_2_	*		0.678502	74.67952	[44]
6	2.73	Dihydrozeatin	220.2	184/167	C_10_H_15_N_5_O	*		0.817118	12.09927	[45]
7	2.79	Gossypetin	317	221/176/96	C_15_H_10_O_8_	*	*	0.712954	71.22651	[46]
8	2.95	Laricitrin	331.1	191/111	C_16_H_12_O_8_	*	*	0.93064	329.4837	[47]
9	3.23	Equol	241.2		C_15_H_14_O_3_		*	0.702379	14.47749	MS-dial
10	9.64	Ellagic acid	301.15	272/165	C_14_H_6_O_8_	*	*	0.931935	19.82557	MS-dial
11	9.76	2'-O-Methyl-5-methyluridine	271.1	–	C_11_H_16_N_2_O_6_		*	0.736028	49.46224	MS-dial
12	10.23	Sucrose	341.1	–	C_12_H_22_O_11_	*	*	0.647778	238.7748	[42]
13	12.21	beta-Hydroxymyristic acid	243.3		C_14_H_28_O_3_		*	0.942568	19.94858	[48]
14	12.77	Estrone	269.2		C_18_H_22_O_2_		*	0.72722	5.531574	[49]
15	12.66	Threonic acid	134.9	–	C_4_H_8_O_5_	*		0.854257	2.89147	[50]
16	13.6	Pantothenic acid	218.15	–			*	0.908312	82.81514	
17	14.24	Inosine-5-monophosphate	347.1		C_10_H_13_N_4_O_8_P	*		0.789652	207.5004	[42]
18	14.38	Malic acid	133.05	–	C_4_H_6_O_5_		*	0.949185	10.3574	[42]
19	14.50	Linoleic acid	279.2	–	C_18_H_32_O_2_		*	0.852049	43.42567	[51]
20	15.20	Quinic acid	191		C_7_H_12_O_6_	*		0.871278	6.95587	[52]
21	15.31	Isorhamnetin	315.1	219/152/108	C_16_H_12_O_7_	*	*	0.726716	26.68773	[53]
22	15.99	6,7-Dihydroxycoumarin	177.15	–	C_9_H_6_O_4_	*		0.690777	16.96198	MS-dial
23	16.26	Adenine	133.9	-	C_5_H_5_N_5_	*		0.805316	7.804921	[42]
24	16.26	Hypoxanthine	134.95	-	C_5_H_4_N_4_O	*	*	0.845876	5.005095	[42]
25	16.45	Retinoic acid	299.3	-	C_20_H_28_O_2_		*	0.837278	36.04725	MS-dial
26	16.55	Kaempferol	285.2	223	C_15_H_10_O_6_	*		0.840564	124.0466	[46]
27	16.89	N-Acetyltryptophan	245	–	C_13_H_14_N_2_O_3_	*		0.652122	41.79542	MS-dial
28	16.82	Docosatetraenoic acid	331.35	–	C_22_H_36_O_2_		*	0.936923	10.88153	MS-dial
29	18.57	Octadecanedioic acid	313.3		C_18_H_34_O_4_	*		0.828433	69.35403	[51]
30	20.48	Heneicosanoic acid	325.3	–	C_21_H_42_O_2_		*	0.789929	16.40839	28]
31	22.18	beta-Bisabolol	221.15	–	C_15_H_26_O	*	*	0.808356	549.4781	[54]
32	22.48	Vergosin	331.3	147	C_16_H_18_O_3_	*	*	0.832863	27.44086	[55]
33	37.03	Deoxyhemigossypol	243.06	153	C_15_H_16_O_3_	*		0.630911	530.6302	[55]
34	38.60	Astragalin	447.11	295/279	C_21_H_20_O_11_	*		0.617828	667.2794	[46]

Ref. mean identification compare to literature and MS-Dial database

In the extraction of industrial waste, there exist certain noteworthy metabolites that are not present in agricultural waste. This disparity may be attributed to the fact that the industrial waste sample contained seeds. Some examples of these distinct metabolites include 2-Hydroxyisobutyric acid, Gluconic acid, Cysteic Acid, Equol, beta-Hydroxymyristic acid, Pantothenic acid, Retinoic acid, Docosatetraenoic acid, and Heneicosanoic acid that are presented in Table 3.

### Antioxidant activity

#### DPPH^.^ scavenging radical

**Fig. 2:**
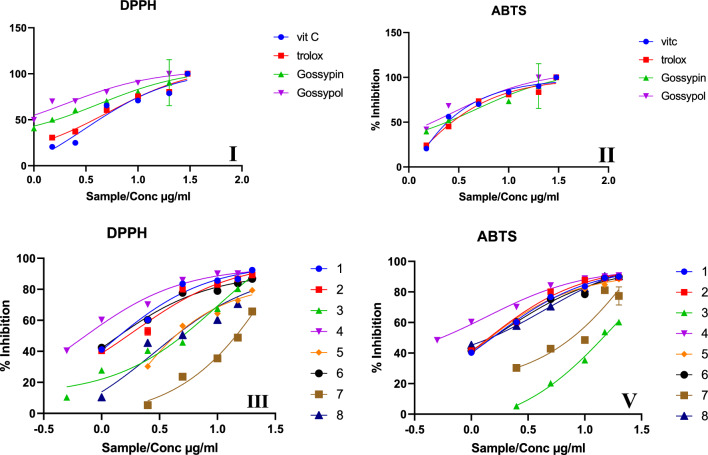
**I** antioxidant activity of Vit C, Trolox, gossypol and gossypin Standard in DPPH $\&$ **II** ABTS. **III** In vitro DPPH^.^ antioxidant activity of different extracts cotton waste as (1 = total agricultural waste, 2 = pet ether fraction, 3 = chloroform fraction, 4 = ethylacetate fraction, 5 = butanol fraction,6 = water fraction, 7 = precipitate_H2O fraction and 8 = total industrial extract) **V** and in ABTS^+.^ Graphs are constructed to show IC_50_ using graph prism

In the present study, results of the antioxidant activity reflected by the DPPH assay has demonstrated that extracts is relatively the best antioxidant active agent when compared with the other sample but still showed semi to lower antioxidant activity than Vit C, Trolox, gossypol, gossypin at the tested concentrations used (Additional file 3: Table (S1) and Fig. (2).

In vitro antioxidant evaluation of total agricultural waste, petroleum ether fraction, chloroform fraction, ethylacetate fraction, butanol fraction, water fraction, pre_H2O fraction and industrial fraction for are presented in Additional file 4: Table (S2). All extracts examined against vitamin C and trolox as a standard. The results revealed that from strong to low IC_50_ gradient as follow 4 > 5 > 1 > 6 > 2 > 8 > 3 > 7 as (IC_50_ 0.6612, 0.8908, 1.056, 1.094, 1.894, 2.892, 10.12, and 42.05, respectively). It was also noticed that the ethylacetate and butanol both extracts showed the highest antioxidant effect than other extracts and four standards through the inhibition of the DPPH free radicals Fig. 2C [56].

#### ABTS ^+^ radical cation

The results give a measure of the antioxidant activity of the ABTS^**.+**^ through measuring the reduction of the radical cation as percentage inhibition of absorbance at 734 nm. Additional file 5: Table (S3) presents the effects of the duration of interaction of specific antioxidants on the suppression of the absorbance of the ABTS^**.+**^ radical cation at 734 nm for trolox, ascorbic acid, gossypin and gossypol the standard reference compounds. The values of the four standards are compared with those of total agricultural waste, pet ether fraction, chloroform fraction, ethylacetate fraction, butanol fraction, water fraction, precipitate_H2O fraction and industrial fraction [56].

The antioxidant activity is concentration dependent on the increases as the concentration increase. This is true with the four standards as used. This pattern is also repeated with all eight samples. Considering the antioxidant activity level from high to low IC_50_ as followed: 5 > 2 > 4 > 1 > 6 > 8 > 3 > 7 as values (0.9706, 1.252, 1.366, 1.519, 1.927, 4.461, 18.18 and 34.78, respectively) Fig. 2D.

The two methods adopted in this part for measuring the antioxidant activity semi confirmed each other, when taking in consideration all fractions. This means that all extracts studied may contain similar chemical group that their effect are attributed to them [26]. In this respect, determination and characterization of such polyphenolics may be useful in developing natural antioxidant substances from a cotton waste material which may add an economic value to beneficial character of the decrease environmental pollution.

### Pharmacological study

#### Acute toxicity study

When the plant extracts were given to animals at a dose of 4 g/kg, neither mortalities nor toxic symptoms were seen. So, they can be utilized safely in accordance to the Organization for Economic Co-operation and Development (OCED) guideline no. 423 (acute class method) because the dose used is larger than 2 g/kg [57]. These findings show that the various cotton extracts are safe and the pharmacological doses were determined accordingly.

#### Investigation of anti-inflammatory activity

The carrageenan rat paw oedema test is one of the most common methods for investigating the anti-inflammatory properties of experimental compounds. Carrageenan produces an inflammatory response that is biphasic. The first stage lasts between one and two and half hours and is characterized by the production of kinin after the first emission of histamine and serotonin. Excessive PGE2 synthesis is the primary mediator of the inflammatory response during the second phase, which lasts for 2.5–6 h [58].

Treatment with gossypin (10 mg/kg), industrial extract (200 mg/kg), and CHCL_3_ extract (100 and 200 mg/kg) significantly decreased paw edema as compared to the control group starting from the first hour after carrageenan injection (Table 4). Their effects were comparable to the indomethacin (standard anti-inflammatory drug). Thus, industrial and chloroform extracts could dose-dependently inhibited the first and second phase of carrageenan-induced paw oedema similar to gossypin treatment, suggesting the inhibition of most inflammatory mediators like histamine, kinin, and PGE_2_. This indicates also the superior anti-inflammatory properties of these agents over the other groups.

**Table 4:** Effect of different cotton extracts and gossypin on percent carrageenan-induced paw edema (E%):

Groups				
	1st h	2nd h	3rd h	4th h
Control	43.55 ± 2.2	48.82 ± 4.56	51.25 ± 3.89	58.66 ± 3.40
Indomethacin (10 mg/kg)	28.01* ± 2.62	37.74* ± 2.35	38.00* ± 3.24	33.56* ± 1.04
Gossypin (10 mg/kg)	29.89* ± 1.98	36.28* ± 3.51	29.13* ± 1.06	27.66* ± 1.92
EtOAc (100 mg/kg)	38.99 ± 3.19	43.57 ± 2.80	49.33 ± 0.86	54.17 ± 2.76
EtOAc (200 mg/kg)	42.66 ± 1.41	48.86 ± 2.2	49.58 ± 1.4	39.63* ± 3.16
Total extract (100 mg/kg)	45.47 ± 1.70	65.96 ± 5.37	66.84 ± 2.03	56.42 ± 4.44
Total extract (200 mg/kg)	40.95 ± 1.55	43.42 ± 2.39	49.52 ± 3.74	50.62 ± 4.35
Industrial 100 mg/kg)	48.76 ± 2.94	49.23 ± 1.39	54.57 ± 3.21	47.51* ± 3.09
Industrial (200 mg/kg)	35.01* ± 1.20	39.80* ± 3.51	40.06* ± 2.04	37.99* ± 2.48
CHCl_3_ (100 mg/kg)	26.27* ± 0.58	35.87* ± 2.44	40.05* ± 1.59	30.67* ± 2.26
CHCl_3_ (200 mg/kg)	27.12* ± 1.62	33.39* ± 1.11	41.72* ± 0.48	29.16* ± 1.06

Every value corresponds to the mean% volume of paw edoema (E%) plus SEM (n = 6). One-way ANOVA was used for statistical analysis, and Tukey's post hoc test was then performed. Changes recorded in paw diameter for each group (at t h) from its parallel basal value (at 0 h) were compared to the control group.* Significantly different from the control group (p < 0.05) at the relevant time interval. *EtOAc*: ethyl acetate; *CHCl3*: chloroform

The EtOAc (200 mg/kg) also induced certain level of anti-inflammatory activity, but, only at the 4th hour after carrageenan injection indicating suppression of prostaglandin release. The anti-inflammatory activity for gossypin was previously stated in literature [59] and was suggested to be due to its cyclooxygenase-2 inhibitory activity. Being a flavone present as a major constituent in the agricultural cotton waste extract and also in the industrial one, gossypin is thought to be responsible for the currently demonstrated anti-inflammatory activity for these extracts.

Gossypol and gossyptin, which are prominent natural polyphenolic compounds derived from cottonseed, have been documented to possess pharmacological properties by modulating the cell cycle and immune signaling pathway. Nevertheless, it remains unclear whether gossypol exhibits anti-inflammatory effects against phytohemagglutinin (PHA)-induced cytokine secretion in T lymphocytes through a similar mechanism. Our study utilizing the T lymphocytes Jurkat cell line demonstrates that exposure to PHA induces a significant increase in the expression of interleukin-2 (IL-2) mRNA and secretion of IL-2. However, these reactions stimulated by PHA are dose-dependently attenuated when pretreated with gossypol. Importantly, gossypol demonstrates no cytotoxic effect in vitro at doses ranging from 5 to 20 μM. In elucidating the possible mechanism underlying the action of gossypol, Chen et al. [60] was observed a pronounced suppression of IL-2 release and a robust decrease in PHA-induced phosphorylation of p38 and c-Jun N-terminal kinase expressions upon gossypol pretreatment. However, no significant phosphorylation of extracellular signal-regulated kinase expression is observed. Additionally, gossypol exhibits the ability to suppress the growth of Jurkat cells, which is associated with an increased percentage of cells in the G1/S phase and a decreased fraction of cells in the G2 phase as determined by flow cytometry. In conclusion, our findings suggest that gossypol exerts anti-inflammatory effects, potentially through partial attenuation of mitogen-activated protein kinase (phosphorylated JNK and p38) signaling and cell cycle arrest in Jurkat cells [61].

#### Effect of cotton extracts on hepatotoxicity induced by CCl_4_

For further verification of the pharmacological activities of the cotton extract fractions, investigation of hepato-protective activity was performed. However, the total extract by its two doses was excluded from this study due to lack of anti-inflammatory effect as shown above and in fulfillment of the reduction principle of the 3R ethical guideline of animal use.

In comparison to the negative control group CCl_4_ injection (p < 0.05) was able to increase serum AST and ALT levels by around 2.5 times [62]. However, when compared to the group that had only received CCl4, the treatment of rats with the industrial extract (200 mg/kg) and CHCl_3_ extract (200 mg/kg) considerably lowered liver enzymes. Both extracts had a similar lowering effect on ALT levels to the standard drug, silymarin, and only CHCl_3_ effect on AST level was equivalent to silymarin. In addition, industrial extract (100 mg/kg) and chloroform extract (100 mg/kg) induced a significant reduction on AST level as compared to CCl_4_-treated group. It is worthy to note that the level of ALT in both silymarin and CHCl_3_ groups was not significantly different from that of the negative control group indicating normalization of ALT level. However, ethyl acetate (EtOAc) extract induced non-significant decrease in liver enzymes as compared to the positive control group (Table 5). Similar hepatoprotective effect of another plant species, *Gossypium hirsutum L.*, was shown by Batur et al. [63]. This cotton honeydew extracts decreased acute liver injury as reflected by decreased transaminases and enhanced liver antioxidant status.

**Table 5:** Effect of different cotton extracts on liver enzymes in CCl4-intoxicated rats:

Groups	AST	ALT
Negative control	93.70^b^ ± 3.71	37.14^b^ ± 3.22
Positive control	237.41^a^ ± 3.62	81.53^a^ ± 4.23
Silymarin (25 mg/kg)	129.80^a,b^ ± 9.08	53.35^b^ ± 3.21
EtOAc (100 mg/kg)	240.30^a^ ± 3.43	75.65^a^ ± 5.67
EtOAc (200 mg/kg)	229.5^a^ ± 13.82	74.30^a^ ± 2.31
Industrial (100 mg/kg)	186.9^a,b^ ± 7.37	70.92^a^ ± 4.93
Industrial (200 mg/kg)	159.61^a,b^ ± 2.66	61.46^a,b^ ± 2.59
CHCl_3_ (100 mg/kg)	160.80^a,b^ ± 2.15	74.30^a^ ± 2.99
CHCl_3_ (200 mg/kg)	131.90^a,b^ ± 4.54	43.89^b^ ± 1.91

Each value represents mean ± SEM (n = 6). Statistical analysis was carried out by One-way ANOVA followed by Tukey post hoc test.

^a^Significantly different form negative control

^b^Significantly different from positive control (CCl_4_) at p < 0.05

##### Antioxidant activity of cotton extracts in CCl4-intoxicated rats

**Fig. 3:**
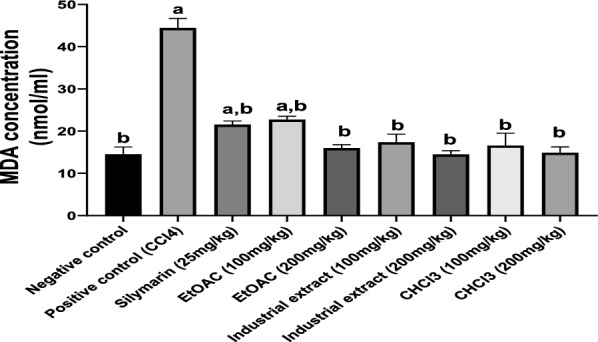
Effect of different cotton extracts on MDA level in CCl_4_-intoxicated rats Each number corresponds to the mean ± SEM (n = 6). One-way ANOVA was used for statistical analysis, followed by the Tukey post hoc test. ^a^significant difference form negative control. ^b^significant difference from positive control (CCl4) at p < 0.05.

CCl_4_ injection induced a significant increase in MDA level (about threefold) when compared to the negative control group indicating increased lipid peroxidation (Fig. 3). Treatment with different cotton extracts decreased MDA significantly as compared to the CCl4 group in a dose-dependent manner. The MDA level in groups treated with EtOAc extract (200 mg/kg), industrial extract (100 and 200 mg/kg), and CHCl_3_ extract (100 and 200 mg/kg) did not differ significantly from that of the normal value and was better than the standard drug, silymarin.

**Fig. 4:**
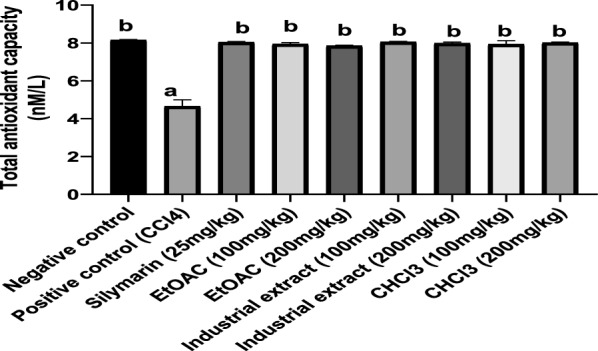
Effect of different cotton extracts on TAC level in CCl_4_-intoxicated rats. Each number corresponds to the mean ± SEM (n = 6). One-way ANOVA was used for statistical analysis, followed by the Tukey post hoc test.^a^significant difference form negative control. ^b^significant differencefrom positive control (CCl4) at p < 0.05. *TAC: total antioxidant capacity*

Additionally, CCl_4_ induced a depression of total antioxidant capacity (TAC) in comparison to the negative control group. However, administration of all investigated cotton extracts significantly enhanced TAC as compared to the CCl4-treated group. The TAC for all extracts was comparable to silymarin (Fig. 4).

By causing irreversible changes to the lipids, proteins, DNA contents and, more importantly, by altering the pathways that regulate normal biological functions, oxidative stress causes hepatic damage and progression of a variety of liver diseases. These include chronic viral hepatitis, alcoholic liver diseases, and non-alcoholic steatohepatitis [64]. In the CCl4-induced liver injury model, oxidative stress is triggered, causing lipid peroxidation to damage the hepatocellular membrane. Subsequently, a significant amount of pro-inflammatory chemokines and cytokines may be released, which may result in more liver injury [65].

Antioxidants have been applied to the treatment of liver disease, thus, showed hepato-protection partially via improving oxidative damage [66]. In the current study, EtOAc, industrial, and CHCl3 extracts of *Gossypium barbadense L* showed better antioxidant activities than the standard drug, silymarin. Similarly*, Gossypium herbaceam* was previously found to possess good antioxidant properties due to flavonoid-rich part and was capable to be used in prevention of Alzheimer disease [53].

Numerous illnesses have been found to originate and progress as a result of oxidative stress. A viable strategy to counteract the negative effects of reactive oxygen species (ROS)-induced oxidative damage is to supplement with exogenous antioxidants or strengthen the body's natural antioxidant defenses. Nearly all of the plant species in the world have outstanding antioxidant potential, and it is thought that two thirds of them have therapeutic significance [67]. Herein, the powerful antioxidant activity of the investigated cotton extracts could effectively illustrates their hepatoprotective effect.

In the current work, the results of in-vivo oxidative stress biomarkers are compatible with the in-vitro antioxidant potential of the different cotton extracts. Both in vitro and in vivo antioxidant activity assessment studies are advisable to be combined in order to assign more precise therapeutic value to plant antioxidant entities [68].

### Effect on the inflammatory marker; C-reactive protein

**Fig. 5:**
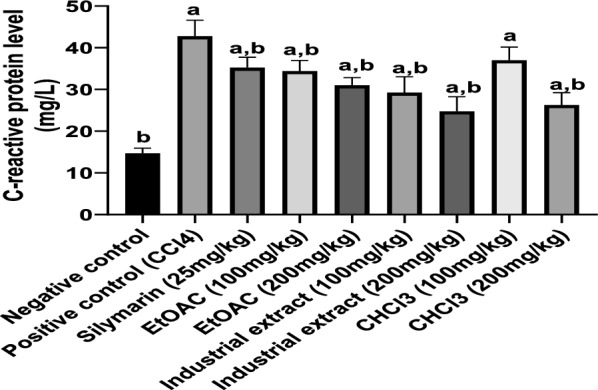
Effect of different cotton extracts on c-reactive protein level in CCl4-intoxicated rats. Each number corresponds to the mean ± SEM (n = 6). One-way ANOVA was used for statistical analysis, followed by the Tukey post hoc test. ^a^significant difference form negative control. ^b^ significant difference from positive control (CCl4) at p < 0.05

CCl4 injection significantly (P < 0.05) increased inflammatory indicator, C-reactive protein, as compared to the negative control group indicating increased lipid peroxidation (Fig. 5**).** Administration of different cotton extracts except CHCl3 (100mg/kg) significantly decreased serum level of C-reactive protein as compared to the CCl4-intoxicated group. Both industrial extract (200 mg/kg) and CHCl3 extract (200 mg/kg) showed the best anti-inflammatory activity which was superior to the standard hepato-protective drug; silymarin.

Hepatic inflammation, which is thought to be the primary cause of hepatic tissue destruction, is a frequent trigger of liver disease. Oxidative stress damage initiates the inflammatory response by activation of the innate immunity. This, in turn, aggravates the consequent processes of fibrosis, cirrhosis [69].

In parallel, the anti-inflammatory activity for other *Gossypium* species was previously shown. *Gossypium herbaceam* could inhibit Nuclear factor kappa-B activity (NF-kappaB) [70]. Another cotton extract flavone component, Gossypetin, also showed anti-inflammatory properties after it had been isolated from the flowers of *Hibiscus sabdariffa* [61]. Gossypin, isolated from *Hibiscus vitifolius* and also present in cotton extract, prevented carcinogenesis progression via its anti-inflammatory properties. It suppressed tumor necrosis factor and NF-kappaB ligand, thus, down-regulated gene products involved in cellular invasion and osteoclastogenesis [71].

### Histopathological examination

**Fig. 6:**
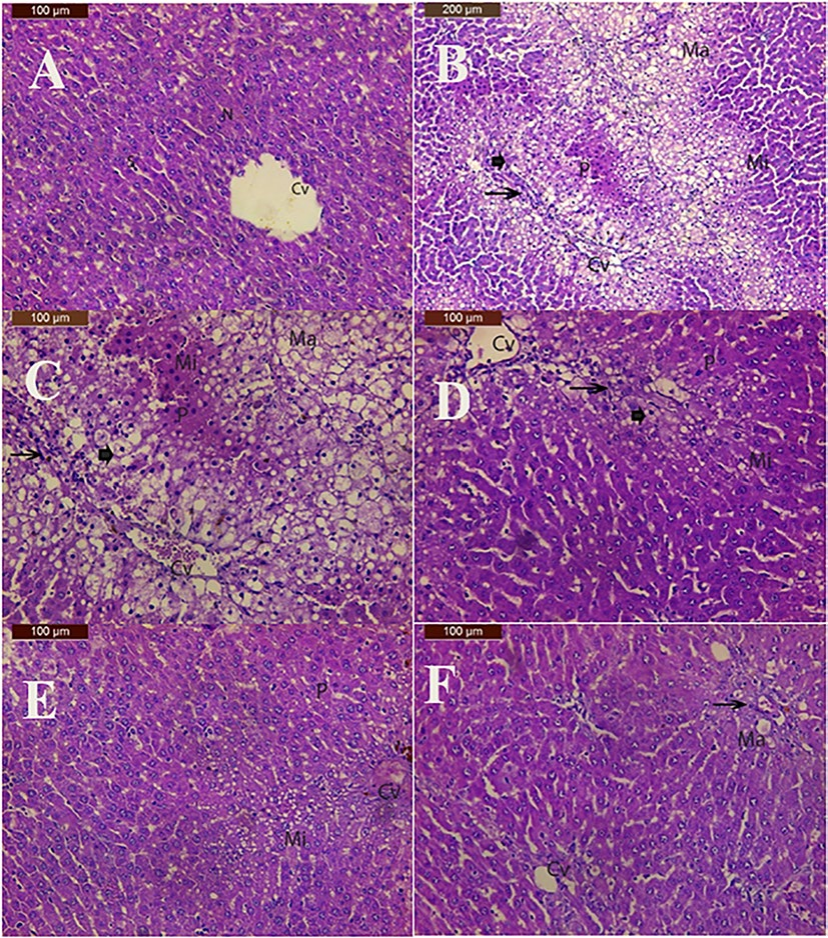
**A**: A photomicrograph of liver of control group showing normal histological architecture with central vein (CV), the hepatic cells with nucleus (N) and the sinusoids (S). **B**: A photomicrograph of liver of CCL_4_ group showing microvesicular (Mi) macrovesicular (Ma) fatty changes of hepatocytes, centrilobular necrosis in most cases, ballooning degeneration (arrowhead) broad mononuclear cells infiltration mostly around central veins and in portal areas with mild fibrous tissues (arrow)., loss of the usual hepatic architecture, deep acidophilic cytoplasm and deeply stained nuclei (P) and many central vein were obviously congested (CV). **C:** High magnification of pervious figure of liver of CCL_4_ group showing microvesicular (Mi) macrovesicular (Ma) fatty changes of hepatocytes, centrilobular necrosis in most cases, ballooning degeneration (arrowhead) broad mononuclear cells infiltration mostly around central veins and in portal areas with mild fibrous tissues (arrow), loss of the usual hepatic architecture, deep acidophilic cytoplasm and deeply stained nuclei (P) and many central vein were obviously congested (CV). **D:** A photomicrograph of liver of CCL_4_ and chloroform extract (100 mg/kg) group showing moderate improvement in hepatic structure with microvesicular fatty changes of hepatocytes (Mi), ballooning degeneration (arrowhead), mild inflammatory cells infiltration (arrow) and pyknotic nuclei (P). **E**: A photomicrograph of liver of CCL_4_ and chloroform extract (200 mg/kg) group showing microvesicular fatty changes of hepatocytes (Mi), ballooning degeneration (arrowhead), mild inflammatory cells infiltration (arrow), congestion of central vein (CV) and pyknotic nuclei (P). **F**: A photomicrograph of liver of CCL_4_ and ethyl acetate extract (100 mg/kg) group showing more or less normal hepatic structure with macrovesicular fatty changes of hepatocytes (Ma) mild inflammatory cells infiltration (arrow)

Sections of the liver from the negative control group showed the hepatocytes' typical architecture to be composed of branching cords that extended outward from the central veins. These cords were separated by blood sinusoids, acidophilic cytoplasm with single central rounded vesicular nuclei (Fig. 6A).

Examination of sections of the rat liver received CCL4 injections for four weeks showed microvesicular and macrovesicular fatty changes of hepatocytes, centrilobular necrosis in most cases, ballooning degeneration and broad mononuclear cells infiltration mostly around central veins and in portal areas with mild fibrous tissues. Also, loss of the usual hepatic architecture, deep acidophilic cytoplasm and deeply stained nuclei and many blood vessels were obviously congested (Fig. 6B, C) similar pathological changes due to CCl4 intoxication were demonstrated in previous studies [72]. Examination of the CCL_4_ and CHCL_3_-extract (100 mg/kg) treated group showed moderate improvement in hepatic structure with microvesicular fatty changes of hepatocytes, mild inflammatory cells infiltration and pyknotic nuclei **(**Fig. 6D**)**. The liver section of CCl_4_ and chloroform extract 200 mg/kg treated group showed ameliorative effect with congestion of central vein, microvesicular fatty changes of hepatocytes, and pyknotic nuclei **(**Fig. 6E**).** In the group treated with CCl_4_ and ethyl acetate extract 100 mg/kg showed more or less normal hepatic structure with microvesicular fatty changes of hepatocytes mild inflammatory cells infiltration **(**Fig. 6F**)**.

**Fig. 7:**
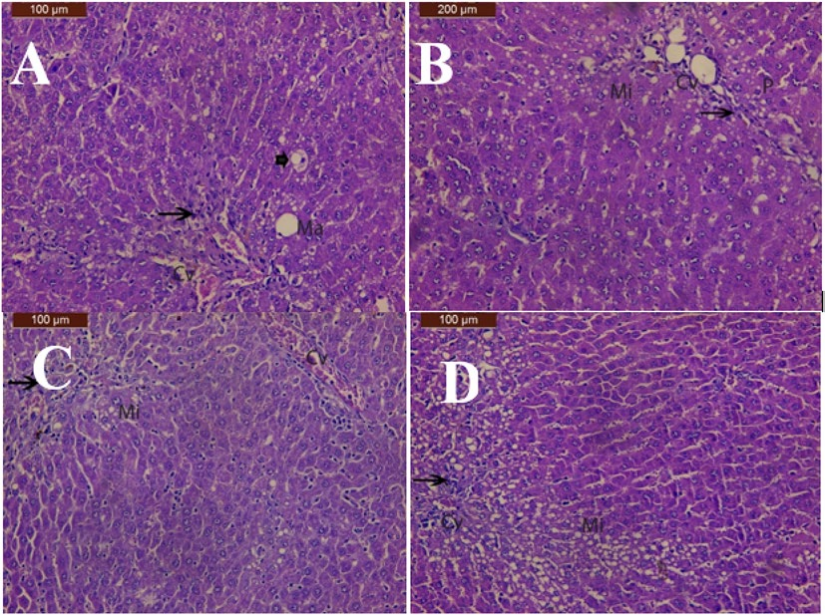
**A** photomicrograph of liver of CCL_4_ and ethyl acetate extract (200 mg/kg) group showing more or less normal hepatic structure with congestion of central vein (CV), macrovesicular fatty changes of hepatocytes (Ma), ballooning degeneration (arrowhead) and mild inflammatory cells infiltration (arrow). **B:** A photomicrograph of liver of CCL_4_ and industrial (100 mg/kg) group showing Liver reduced the development of histopathological damage except microvesicular fatty changes of hepatocytes (Mi) mild inflammatory cells infiltration (arrow) and pyknotic nuclei (P). **C** A photomicrograph of liver of CCL_4_ and industrial (200 mg/kg) group showing Liver decrease the development of histopathological damage except congestion of central vein (CV), microvesicular fatty changes of hepatocytes (Mi) and pyknotic nuclei (P). **D:** A photomicrograph of liver of CCL_4_ and silymarin group showing moderate improvement with less inflammation and less ballooning and moderate microvesicular fatty changes of hepatocytes (Mi)

In the group treated with CCl_4_ and ethyl acetate extract (200 mg/kg) showed more or less normal hepatic structure with congestion of central vein, macrovesicular fatty changes of hepatocytes, ballooning degeneration and mild inflammatory cells infiltration **(**Fig. 7A**)**. Liver sections of CCL_4_ with industrial (100 mg/kg) group showed reduced the development of histopathological damage except microvesicular fatty changes of hepatocytes mild inflammatory cells infiltrationand pyknotic nuclei (Fig. 7B). Liver sections of CCL_4_ with industrial (200 mg/kg) group showed improved hepatic structure except little congestion of central vein, microvesicular fatty changes of hepatocytes and pyknotic nuclei (Fig. 7C). Silymarin and CCL_4_ group section showed moderate improvement with less inflammation and less ballooning and moderate microvesicular fatty changes of hepatocytes was observed (Fig. 7D).

In consistence with the biochemical results, the histopathological finding confirmed the hepatoprotection exhibited by EtOA_C_, CHCl_3_, and industrial cotton waste extracts. The reduction in hepatic injury was superior in rats treated by CHCl_3_, and industrial cotton waste extracts at high doses. The hepatoprotective activity was exerted in a dose-dependent manner.

## Conclusion

Comparative analysis of gossypin and gossypol content was performed on different fractions of cotton solid waste extracts. The results indicated ethyl acetate containing higher gossypin levels than butanol fraction. Gossypol was also found with greater amounts in chloroform rather than petroleum ether. The choice of the proper solvents; CHCL_3_ in case of gossypol and EtOAc in case of gossypin may depend on the solubility desired by their phenolic nature. Chloroform extract and industrial waste extract showed superior anti-inflammatory and hepatoprotective effects against CCl4 induced hepatotoxicity in a dose-dependent manner, as demonstrated by the biochemical indicators’ considerable recovery. Ethyl acetate, chloroform, and industrial waste extracts showed proper antioxidant activities. The hepatoprotective efficacy of these extracts was further supported by the histopathological analysis. The presence of the flavonoid gossypin, as well as the other active ingredients, is thought to be associated with the hepatoprotective effect of cotton waste extracts. The extraction of bioactive substances with biological activity using the proper extraction method as demonstrated by the present work could help in green economy due to the commercial value of the outcome. Besides, this may contribute to climate change mitigation efforts as it introduces an ecofriendly alternative way of disposal of cotton solid wastes both in the farm and in industry.

### Supplementary Information

**Additional file 1: Fig S1.** HPLC of standard Gossypin and Gossypol.

**Additional file 2: Fig S2.** Determination of gossypin and gossypol by HPLC in eight samples.

**Additional file 3: Table S1.** In vitro ABTS+ and DPPH antioxidant activity of Standards.

**Additional file 4: Table S2.** In vitro DPPH antioxidant activity of different extracts cotton waste.

**Additional file 5: Table S3.** In vitro ABTS^+^ antioxidant activity of different extracts cotton waste.

## Data Availability

The datasets used during the current study available from the corresponding author on reasonable request.

## Abbreviations

HPLC: High performance liquid chromatography
LC–ESI–MS/MS: Liquid chromatography–electrospray ionization–tandem mass spectrometry
ESI: Electrospray ionization
EtOAc: Ethyl acetate
CHCl3: Chloroform
TAC: Total antioxidant capacity.
DPPH: 2,2-Diphenylpicrylhydrazyl
ABTS: 2,2-Azino-bis-3-ethylbenzothiazoline-6-sulfonic acid
CCl4: Carbon tetrachloride
NF-kappa B: Nuclear Factor-kappa B activity
