# Supplementary figures and images for: A comprehensive tool in recycling plant-waste of Gossypium barbadense L agricultural and industrial waste extracts containing gossypin and gossypol: hepatoprotective, anti-inflammatory and antioxidant effects
Source: Plant Methods. 2024 Apr 17;20:54. doi: 10.1186/s13007-024-01181-8 (PMC11022478; doi:10.1186/s13007-024-01181-8)

| **STD** | | |
| --- | --- | --- |
|  | **ConC. µg/ml** | **Area** |
| **Gossypin** | **10** | **430.66** |
| **Gossypol** | **20** | **420.01** |


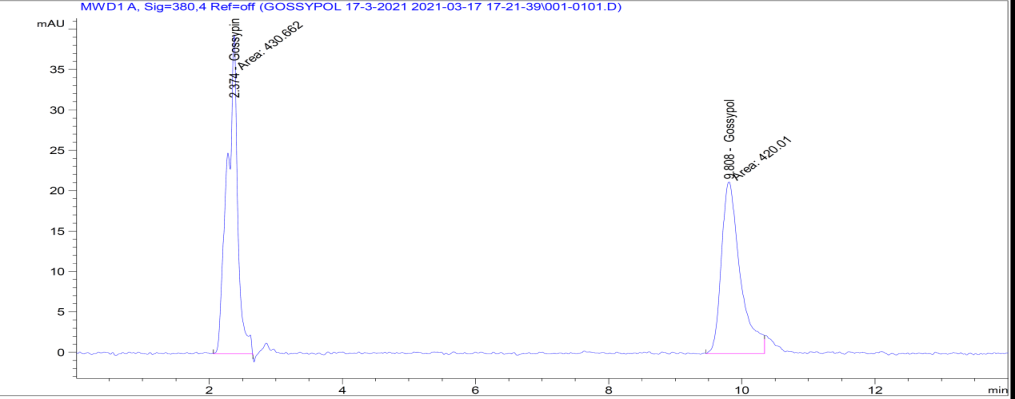


**Fig S1 HPLC of standard Gossypin and Gossypol.**

Supplement: Supplementary file 1 — Additional file 1: Fig S1. HPLC of standard Gossypin and Gossypol. [file 13007_2024_1181_MOESM1_ESM.docx]
