# Supplementary material for: A comprehensive tool in recycling plant-waste of Gossypium barbadense L agricultural and industrial waste extracts containing gossypin and gossypol: hepatoprotective, anti-inflammatory and antioxidant effects
Source: Plant Methods. 2024 Apr 17;20:54. doi: 10.1186/s13007-024-01181-8 (PMC11022478; doi:10.1186/s13007-024-01181-8)
Supplement: Supplementary file 2 — Additional file 2: Fig S2. Determination of gossypin and gossypol by HPLC in eight samples. [file 13007_2024_1181_MOESM2_ESM.docx]

Total Agricultural _waste


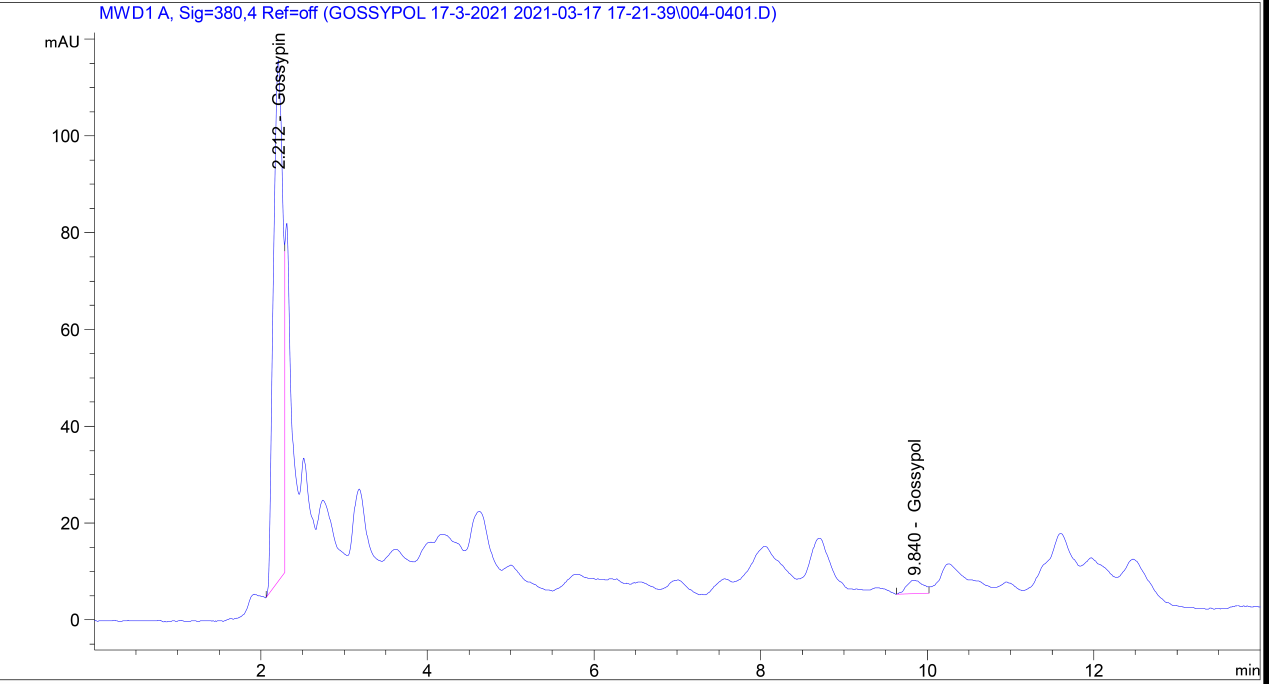


Pet. Ether_ Fraction


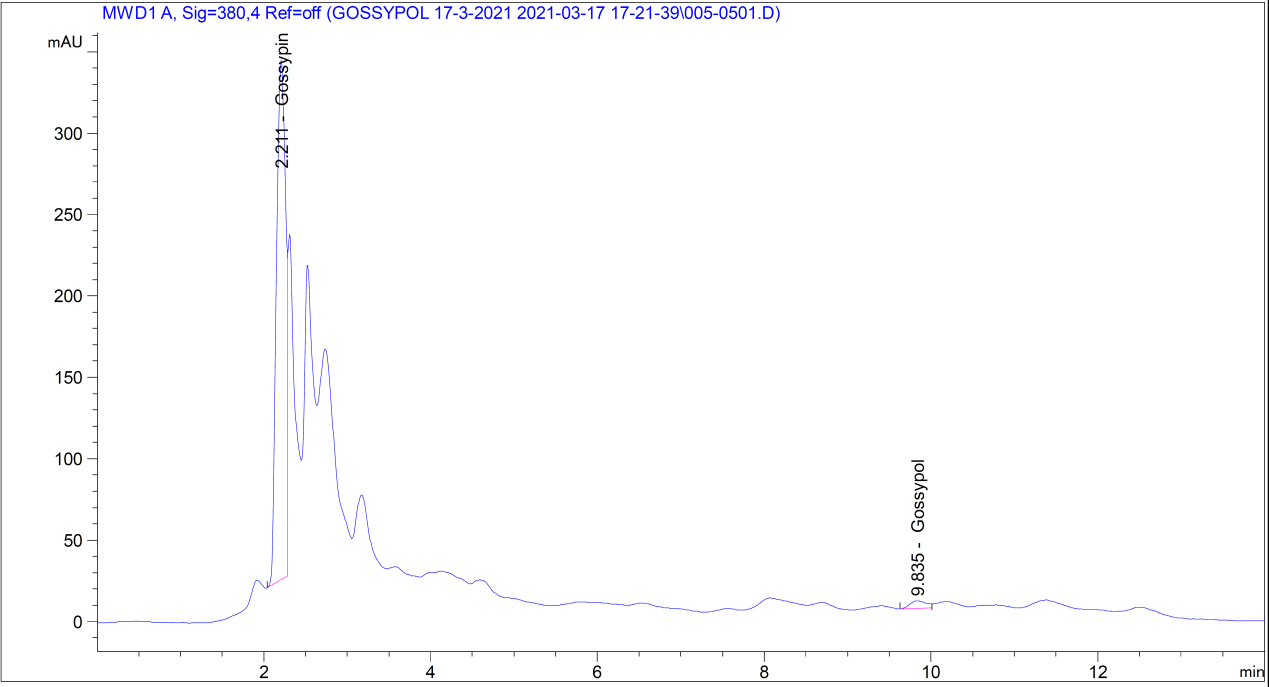


CH_2_CL_2__ Fraction

EtOAc_Fraction

_ fraction

BuOH_Fraction

_ fraction

H_2_O_Fraction

_ fraction

Pre_H_2_O_Fraction

_ fraction

Industrial waste

_ fraction

**Fig S2 Determination of gossypin and gossypol by HPLC in eight samples.**
