# Supplementary material for: A comprehensive tool in recycling plant-waste of Gossypium barbadense L agricultural and industrial waste extracts containing gossypin and gossypol: hepatoprotective, anti-inflammatory and antioxidant effects
Source: Plant Methods. 2024 Apr 17;20:54. doi: 10.1186/s13007-024-01181-8 (PMC11022478; doi:10.1186/s13007-024-01181-8)
Supplement: Supplementary file 3 — Additional file 3: Table S1. In vitro ABTS+ and DPPH antioxidant activity of Standards. [file 13007_2024_1181_MOESM3_ESM.docx]

**Table S*1. In vitro* ABTS+ and DPPH antioxidant activity of Standards.**

| **Sample/ Conc µg/ml** | **DPPH** | | | | **ABTS** | | | |
| --- | --- | --- | --- | --- | --- | --- | --- | --- |
|  | **vit C** | **trolox** | **Gossypin** | **Gossypol** | **vitc** | **trolox** | **Gossypin** | **Gossypol** |
| 30 | 100 ± 0.00 | 100 ± 0.00 | 100 ±0.00 | 100 ± 0.0 | 100 ± 0.00 | 100 ± 0.00 | 100 ± 0.00 | 100 ± 0.02 |
| 20 | 78.80 ± 0.56 | 80.35 ± 0.52 | 90.25 ± 0.25 | 100 ± 0.0 | 90.23 ± 0.85 | 83.55 ± 0.81 | 90.25 ± 0.25 | 100 ± 0.23 |
| 10 | 70.91 ± 0.89 | 75.58 ± 0.75 | 80.26 ± 0.21 | 90.25 ± 0.12 | 83.63 ± 0.86 | 80.77 ± 1.02 | 73.26 ± 0.54 | 82.25 ± 0.25 |
| 5 | 65.62 ± 0.94 | 60.59 ± 0.85 | 70.26 ± 0.54 | 80.26 ± 0.09 | 40.03 ± 0.99 | 63.42 ± 0.85 | 71.32 ± 0.69 | 70.26 ± 0.15 |
| 2.5 | 24.93 ± 0.33 | 37.20 ± 0.42 | 60.32 ± 0.69 | 70.26 ± 0.25 | 26.19 ± 0.25 | 45.21 ± 0.89 | 52.00 ± 0.25 | 68.25 ± 0.02 |
| 1.5 | 20.51 ± 0.52 | 30.55 ± 0.96 | 50 ± 0.25 | 70.32 ± 0.15 | 20.52 ± 0.65 | 24.20 ± 0.85 | 39.56 ± 0.12 | 42.00 ± 0.25 |
| 1 | - | - | 40.56 ±0.12 | 50.23 ± 0.45 | - | - | - | - |
| IC50 | 3.078 | 5.168 | 4.249 | 2.024 | 0.1107 | 0.7652 | 4.728 | 2.738 |
