# Supplementary material for: A comprehensive tool in recycling plant-waste of Gossypium barbadense L agricultural and industrial waste extracts containing gossypin and gossypol: hepatoprotective, anti-inflammatory and antioxidant effects
Source: Plant Methods. 2024 Apr 17;20:54. doi: 10.1186/s13007-024-01181-8 (PMC11022478; doi:10.1186/s13007-024-01181-8)
Supplement: Supplementary file 4 — Additional file 4: Table S2. In vitro DPPH antioxidant activity of different extracts cotton waste. [file 13007_2024_1181_MOESM4_ESM.docx]

**Table S2.  *In vitro* DPPH antioxidant activity of different extracts cotton waste.**

| **DPPH/Conc**  **Ug/ml** | **1** | **2** | **3** | **4** | **5** | **6** | **7** | **8** |
| --- | --- | --- | --- | --- | --- | --- | --- | --- |
| 20 | 92.36 ± 0.07 | 89.190± 0.05 | 90.23 ± 0.05 | 90.44 ± 0.070 | 79.37 ± 0.070 | 86.70 ± 0.20 | 65.68 ± 0.07 | 89.79 ± 0.18 |
| 15 | 86.52 ± 0.04 | 86.70 ± 0.00 | 80.32 ± 0.04 | 90.20 ±0.07 | 72.82 ± 0.07 | 83.85 ±0.00 | 48.84 ± 0.07 | 70.26 ± 0.14 |
| 10 | 85.62 ± 0.08 | 83.16 ±0.08 | 67.45 ± 0.07 | 90.10 ± 0.00 | 64.40 ± 0.12 | 78.92 ± 0.08 | 35.41 ± 0.14 | 60.56 ±0.07 |
| 5 | 83.50 ± 0.08 | 80.06± 0.08 | 45.74 ± 0.01 | 85.91± 0.08 | 56.47± 0.08 | 77.69± 0.01 | 23.58± 0.00 | 50.35 ± 0.16 |
| 2.5 | 60.28 ± 0.06 | 52.86± 2.46 | 40.42 ± 0.05 | 70.21± 0.07 | 30.25± 0.01 | 60.27± 0.04 | 5.27± 0.04 | 45.70 ± 0.37 |
| 1 | 41.24± 0.01 | 40.25± 0.02 | 27.72± 0.08 | 60.29± 0.05 | - | 42.33± 0.02 | - | 35.26± 0.05 |
| 0.5 | - | - | 10.28± 0.06 | 40.42 ± 0.08 | - | - | - | - |
| IC50 | 1.056 | 1.894 | 10.12 | 0.6612 | 0.8908 | 1.094 | 42.05 | 2.892 |

- 1=total agricultural waste , 2=pet ether fraction, 3=dichloromethane fraction, 4=ethylacetate fraction, 5=butanol fraction,6= water fraction, 7=pre_H2O fraction and 8=industrial fraction
