# Supplementary material for: A comprehensive tool in recycling plant-waste of Gossypium barbadense L agricultural and industrial waste extracts containing gossypin and gossypol: hepatoprotective, anti-inflammatory and antioxidant effects
Source: Plant Methods. 2024 Apr 17;20:54. doi: 10.1186/s13007-024-01181-8 (PMC11022478; doi:10.1186/s13007-024-01181-8)
Supplement: Supplementary file 5 — Additional file 5: Table S3. In vitro ABTS+ antioxidant activity of different extracts cotton waste. [file 13007_2024_1181_MOESM5_ESM.docx]

**Table S3.  *In vitro* ABTS^+^ antioxidant activity of different extracts cotton waste.**

| **ABTS/Conc**  **Ug/ml** | **1** | **2** | **3** | **4** | **5** | **6** | **7** | **8** |
| --- | --- | --- | --- | --- | --- | --- | --- | --- |
| 20 | 90.06 ± 0.04 | 89.56 ± 0.13 | 60.24 ± 0.01 | 90.76 ± 0.13 | 88.33 ± 0.04 | 89.84 ± 0.47 | 77.35 ± 4.84 | 90.21 ± 0.13 |
| 15 | 89.20 ± 0.01 | 88.92 ± 0.01 | 53.68 ± 0.27 | 90.12 ± 0.13 | 84.76 ± 0.23 | 89.29 ± 0.01 | 81.16 ± 0.01 | 90.21± 0.13 |
| 10 | 83.65 ± 0.01 | 87.81 ± 0.27 | 35.28 ± 0.42 | 88.82 ± 0.32 | 81.99± 0.01 | 78.44 ± 0.32 | 48.56± 0.89 | 82.17 ± 0.16 |
| 5 | 76.74 ± 0.02 | 80.22 ± 0.06 | 20.24 ± 0.03 | 84.30 ± 0.16 | 79.04 ± 0.16 | 75.26 ± 0.01 | 42.84 ± 0.42 | 70.35 ± 0.16 |
| 2.5 | 60.25 ± 0.01 | 60.28 ± 0.04 | 5.18 ± 0.11 | 70.21± 0.08 | 40.24 ± 0.03 | 60.27 ± 0.05 | 30.25 ± 0.01 | 57.71 ± 0.37 |
| 1 | 40.25 ± 0.01 | 41.62 ± 0.60 | - | 60.37 ± 0.15 | 40.24 ± 0.02 | 41.538 1.35 | - | 45.31± 0.05 |
| 0.5 | - | - | - | - | - | 48.48 ± 0.42 | - | - |
| IC50 | 1.519 | 1.252 | 18.18 | 1.366 | 0.9706 | 1.927 | 34.78 | 4.461 |

1=total agricultural waste , 2=pet ether fraction
